# High-Performance Motion Estimation for Image Sensors with Video Compression

**DOI:** 10.3390/s150820752

**Published:** 2015-08-21

**Authors:** Weizhi Xu, Shouyi Yin, Leibo Liu, Zhiyong Liu, Shaojun Wei

**Affiliations:** 1 Institute of Microelectronics, Tsinghua University, Beijing 100084, China; E-Mails: weizhixu@gmail.com (W.X.); liulb@tsinghua.edu.cn (L.L.); wsj@tsinghua.edu.cn (S.W.); 2 School of Information Science and Engineering, Shandong Normal University, Jinan 250014, China; 3 State Key Laboratory for Computer Architecture, Institute of Computing Technology, Chinese Academy of Sciences, Beijing 100190, China; E-Mail: zyliu@ict.ac.cn

**Keywords:** memory bandwidth, image sensors, video compression, motion estimation, full search, inter-frame, data reuse

## Abstract

It is important to reduce the time cost of video compression for image sensors in video sensor network. Motion estimation (ME) is the most time-consuming part in video compression. Previous work on ME exploited intra-frame data reuse in a reference frame to improve the time efficiency but neglected inter-frame data reuse. We propose a novel inter-frame data reuse scheme which can exploit both intra-frame and inter-frame data reuse for ME in video compression (VC-ME). Pixels of reconstructed frames are kept on-chip until they are used by the next current frame to avoid off-chip memory access. On-chip buffers with smart schedules of data access are designed to perform the new data reuse scheme. Three levels of the proposed inter-frame data reuse scheme are presented and analyzed. They give different choices with tradeoff between off-chip bandwidth requirement and on-chip memory size. All three levels have better data reuse efficiency than their intra-frame counterparts, so off-chip memory traffic is reduced effectively. Comparing the new inter-frame data reuse scheme with the traditional intra-frame data reuse scheme, the memory traffic can be reduced by 50% for VC-ME.

## 1. Introduction

Video compression (VC) is very widely used, e.g., in mobile phones, notebook computers, video sensor networks (VSN), and so on. Due to the ease of deployment, dynamically-configurable nature and self-organizing characteristics, VSN becomes more and more popular [1]. VSNs are essential in the surveillance of remote areas, such as monitoring of battlefields, farmlands, and forests. VSNs can also be used in intelligent transportation, environmental monitoring, public security, and so on. An important factor for VSN is the transmission speed of the video contents for real-time processing. The video contents obtained by image sensors in VSN are usually compressed to reduce the time overhead of video transmissions. Therefore, it is also important to reduce the time overhead for video compression or its kernel algorithm motion estimation (ME).

**Figure 1:**
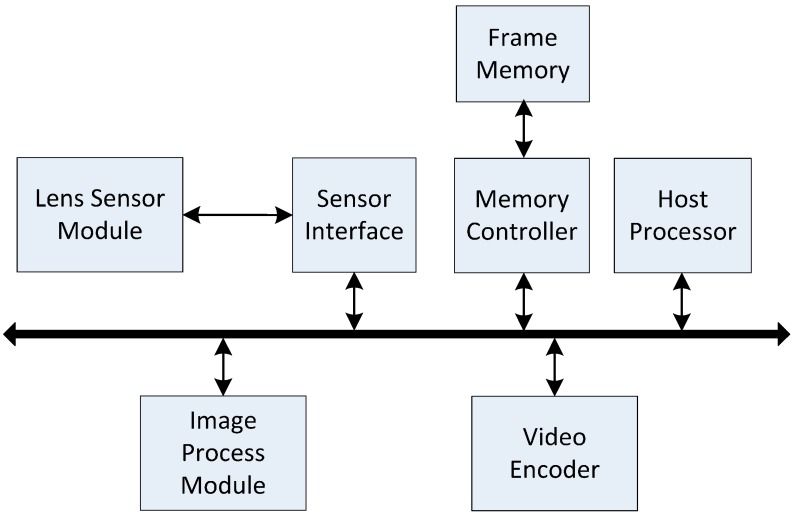
Block diagram of a video-acquisition system.

Figure 1 gives the block diagram of a video-acquisition system [2]. The sensor interface obtains pixels from the image sensor (Lens Sensor Module) and continuously transfers them to the external memory (Frame Memory) via the memory controller. The type of the external memory is DDR3 memory with 64-bits bus width, 4GB capacity and 800 MHz memory clock frequency. Once one row of pixel-blocks in an image is ready in the external memory, the image process module processes these pixel-blocks one by one. The output pixel-block can be transferred to a typical video encoder module for video compression. The host processor (e.g., an ARM processor) is used to control or configure the other modules.

**Figure 2:**
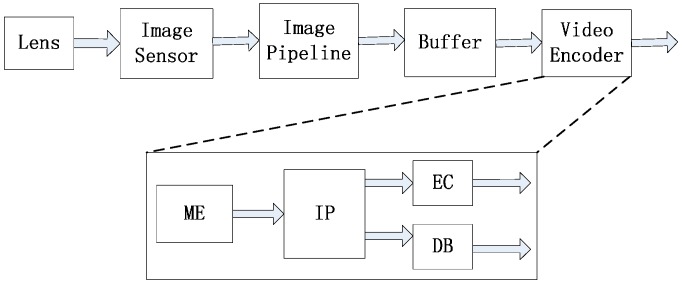
Data flow for a video-acquisition system mainly including an image sensor, an image processing pipeline and a video encoder for video compression.

Figure 2 shows the data flow for a video-acquisition system mainly including an image sensor with video compression. The lens and image sensor are used to acquire the raw image. The image pipeline processes the pixels from the image sensor to restore vivid videos. A buffer is used to reorder line scan pixels to block scan ones. The video encoder compresses the video from the buffer and output the compressed Bitstream for transmission. In the video encoder, ME is used to reduce temporal redundancy among adjacent frames for video compression (VC-ME) [3].

There are many ME implementations on various platforms (embedded systems, GPU and FPGA) for different types of ME, such as gradient-based, energy-based and block-based [4,5,6,7,8,9,10,11,12,13]. There are a lot of works for accelerating ME based on specific hardware. A novel customizable architecture of a neuromorphic robust optical flow (multichannel gradient model) is proposed in [4], which is based on reconfigurable hardware with the properties of the cortical motion pathway. A complete quantization study of neuromorphic robust optical flow architecture is performed in [5], using properties found in the cortical motion pathway. This architecture is designed for VLSI systems. An extensive analysis is performed to avoid compromising the viability and the robustness of the final system. A robust gradient-based optical flow model is efficiently implemented in a low-power platform based on a multi-core digital signal processor (DSP) [6]. Graphics processor units (GPUs) offer high performance and power efficiency for a large number of data-parallel applications. Therefore, there are also many works using GPU or multi-GPU to accelerate motion estimation [7,8]. FPGA is also a very useful platform to accelerate motion estimation [9,10]. There is also a novel bioinspired sensor based on the synergy between optical flow and orthogonal variant moments; the bioinspired sensor has been designed for VLSI and implemented on FPGA [9]. A tensor-based optical flow algorithm is developed and implemented using field programmable gate array (FPGA) technology [10].

Block-based ME is popular for its simplicity and efficiency, and there are also many hardware-based block-matching algorithms [11,12,13]. Block matching is used to find the best matching macro-block (MB) in the reference frame with the current MB. The sum of absolute differences (SAD) is one way to determine the best match. The displacement between the current MB and the best matching reference MB is the motion vector (MV). ME usually takes most of the time in VC and the accuracy of ME affects the compression ratio in VC. Full search integer ME (FSIME) employs brute-force search to find the optimal MB in the search range and achieves the best accuracy. FSIME is suitable for efficient hardware implementation because of its regularity but it demands a large number of computations and memory accesses. Therefore, it is important to reduce the time cost of VC-ME in VSN. Fast search methods are proposed to reduce time overhead usually with loss of accuracy, such as Three Step Search [14], New Three Step Search [15], Diamond Search [16,17] and Four Step Search [18]. Fast search methods may not find the optimal MB and many of them are not regular for hardware implementation. We focus on FSIME in this paper.

In recent years, the speed gap between on-chip computing and off-chip memory access has grown larger and larger, so it is important to reduce off-chip bandwidth requirement to improve overall performance especially for real-time video applications [19]. Reusing data on chip is usually considered to reduce off-chip memory traffic. Some data reuse methods are proposed for FSIME [20,21,22,23,24]. Previous work mainly focused on intra-frame data reuse within a reference frame but inter-frame data reuse was neglected. For VC-ME, the reconstructed frame was usually stored to off-chip memory and then loaded to on-chip memory when needed [23,25,26,27,28]. If the reconstructed frame is reused on chip without being stored to off-chip memory for VC-ME, the off-chip memory traffic will be greatly reduced.

In this paper, we propose a high-performance motion estimation to reduce the time cost for image sensors with video compression. The new method exploits inter-frame data reuse with affordable on-chip memory size for FSIME. The inter-frame data reuse scheme can effectively reduce off-chip memory traffic. For VC-ME, reconstructed frames are stored on chip until it is used by the next current frame. Three levels (Inter-E, Inter-D, Inter-C) of the proposed inter-frame data reuse scheme are presented and analyzed, which gives a good tradeoff between data reuse efficiency and on-chip memory size. All the three levels have better data reuse efficiency than their intra-frame counterparts. Furthermore, the new method is compatible with different intra-frame data reuse schemes [20,21] and scan orders [22,23]. Comparing the inter-frame data reuse scheme with the intra-frame data reuse scheme, we find that the memory traffic can be reduced by 50% for VC-ME according to the case study. 

The rest of the paper is organized as follows: the parallelism and data locality of FSIME are analyzed in Section 2; three levels of inter-frame data reuse scheme for VC-ME are proposed and analyzed in Section 3; an implementation of the inter-frame data reuse architecture is presented in Section 4; experiment results are given in Section 5; and Section 6 is the conclusion.

## 2. Parallelism and Locality Analysis for FSIME

Some basic concepts are explained in Table 1 for better understanding of the following sections. MB, BS, SR, and SRS are four levels of data ranges in a frame. Different scan orders are used to implement different data reuse schemes and can lead to different data reuse efficiency [22,23].

**Table 1:** Basic concepts.

Concept	Explanation
Marco Block(MB)	A block of pixels in a frame with size of N × N, 16 × 16 for H.264, 64 × 64 for HEVC
Current Block(CB)	A marco block in the current frame
Block Strip(BS)	A row of MBs in a frame
Search Range(SR)	Search range in reference frame for current block
SR Strip(SRS)	A row of search ranges
SR_V_ and SR_H_	Vertical and Horizontal search range
W and H	Width and Height of a frame
Scan Orders	Raster scan, snake scan, smart snake scan, OSP
Processing Element Array (PEA)	The IME engine to compute the SAD value and MV

### 2.1. Parallelism Analysis for FSIME

**Figure 3:**
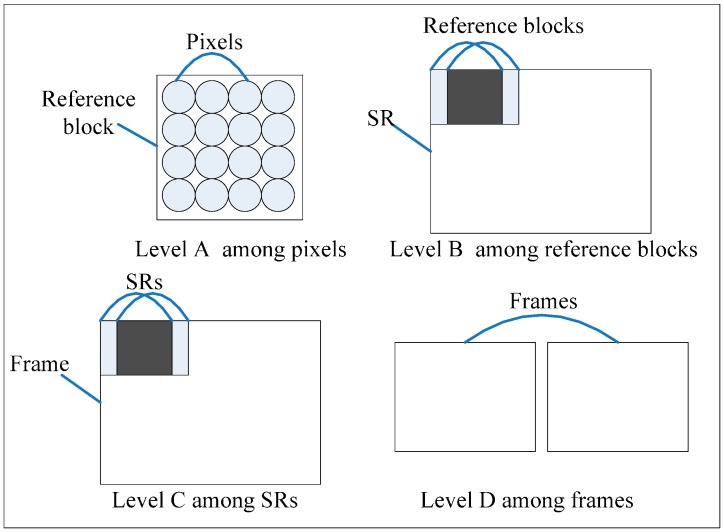
Four different levels of parallelism for FSIME.

There are four levels of parallelism for FSIME (Figure 3). Level A is the parallelism among pixels. Different pixels can be computed in parallel to get SAD between two MBs. A typical implementation of Level A is an *N × N* PEA with an adder tree [25]. Level B is the parallelism among reference blocks. One CB is compared with all the reference blocks in SR, and different reference blocks can be computed in parallel [26]. Level C is the parallelism among SRs or CBs. Different SRs in a frame can be computed in parallel [28]. Level C cannot be directly applied to VC-ME because of data dependency between adjacent CBs. After modifying the method of computing the MV predictor [25], data dependency is eliminated and Level C can be applied to VC-ME. Level D is the parallelism among frames. For VC-ME, the current frame cannot be processed until the previous frame is reconstructed so Level D is not possible. The parallelism degrees for the four parallelism levels are listed in Table 2, and it is assumed that there is one reference frame for a current frame. F is the number of the current frames, and it is assumed that there are F frames in the video sequence.

**Table 2:** Different Parallelism Levels.

Level	Parallelism Explanation	Max. Degree of Parallelism
Level A	among pixels	F × W × H × SR_H_ × SR_V_
Level B	among reference blocks	F × (W/N) × (H/N) × SR_H_ × SR_V_
Level C	among SRs	F × (W/N) × (H/N)
Level D	among frames	F

### 2.2. Data Locality Analysis for FSIME

Data locality includes spatial locality and temporal locality. A common technique to utilize spatial locality is pre-fetching. The temporal locality can be analyzed for better implementation of data reuse, which is our focus. Different intra-frame data reuse levels are proposed and analyzed in Table 3. Level A is within a reference block strip in an SR. Level B is among adjacent reference block strips in an SR. Level C is within SRS, and is the most popular data reuse level. Level D is among adjacent SRSs, which is a one-access data reuse level [20,29]. Level C+ [21] is the data reuse level between Level C and D, which elaborately employs tradeoffs between data reuse efficiency and on-chip memory size. For variable block size ME, SAD of smaller blocks can be reused to compute SAD of larger blocks. That is the data reuse within one MB, and we call it Level O data reuse. Level A+ is the data reuse level between Level A and B, which also employs tradeoff between data reuse efficiency and on-chip memory size. Level A+ is similar to Level C+. However, Level A+ is usually implemented to load data from SRAM to registers [22] while Level C+ is often used to load data from off-chip memory to on-chip memory.

The above data reuse levels only consider the data reuse within the reference frame, and we call them intra-frame data reuse levels. The disadvantage of only using intra-frame data reuse is that the reconstructed frame is usually stored to off-chip memory and then loaded to on-chip memory when needed for VC-ME. Level E is a type of inter-frame reuse level, but it was not analyzed in detail and considered to be impractical because it demanded storing at least one frame on chip [20]. 

Traditionally, Ra, defined by Equation (1), is the redundancy access factor which is used to evaluate memory accesses efficiency [20]. Only the memory traffic of loading the reference frame is considered for Ra. We list Ra and on-chip memory size of intra-frame data reuse levels in Table 3. n is the parameter of C+ scheme.

(1) $$Ra=\frac{memory\;accesses\;for\;loading\;reference\;frame}{pixel\;count\;of\;a\;frame}$$

**Table 3:** Intra-frame Data Reuse Levels.

Reuse Level	Ra	On-Chip Memory Size
No reuse	SR_V_ × SR_H_	0
Intra-A	SR_V_(1 + SR_H_/N)	N × (N−1)
Intra-B	(1 + SR_V_/N) (1 + SR_H_/N)	(N + SR_H_) × (N−1)
Intra-C	1 + SR_V_/N	(SR_H_ + N−1) × (SR_V_ + N−1)
Intra-C+	1 + SR_V_/nN	(SR_H_ + N−1) × (SR_V_ + nN−1)
Intra-D	1	(SR_H_ + W−1) × (SR_V_−1)

## 3. Inter-Frame Data Reuse Scheme for VC-ME

VC-ME aims to reduce the temporal redundancy in adjacent frames. The reconstructed frame instead of original frame is used as the reference frame for VC-ME. In this section, we present and analyze three inter-frame data reuse levels for two kinds of VC-ME, single reference frame VC-ME (VC-SRME) and multiple reference frames VC-ME (VC-MRME) [30].

### 3.1. New Definition of Ra for VC-ME

Only the memory traffic of loading the reference frame was considered in previous work [20,21,22,23] when computing Ra. However, the memory traffic for storing the reconstructed frame to off-chip memory and loading the current frame from off-chip memory should also be considered. We use Equation (2) as the new definition of Ra for VC-ME. The memory traffic of loading the reference frame (Ra_intra) is computed by Equation (3), where memref_load_ is the memory traffic to load a reference frame. Ra_inter defined in Equation (4) rises from storing reconstructed frame to off-chip memory (memref_store_) and loading current frame to on-chip memory (memcur_load_).

(2) Ra=Ra_intra+Ra_inter

(3) $$Ra\_intra=\frac{memref_{load}}{pixel\;count\;of\;a\;frame}$$

(4) $$Ra\_inter=\frac{memref_{store}+memcur_{load}}{pixel\;count\;of\;a\;frame}$$

Ra of different intra-frame data reuse levels for VC-SRME (Table 4) can be calculated according to Equations (2)–(4). For example, Ra of Intra-C is calculated as follows:

$$(1+SR_{V}/N)+(W\times H+W\times H)/(W\times H)\;=(1+SR_{V}/N)+2$$

**Table 4:** Ra and On-chip Memory Size for VC-SRME.

Level	Ra	On-Chip Memory Size
No reuse	SR_V_ × SR_H_ + 2	0
Intra-A	SR_V_(1 + SR_H_/N) + 2	N × (N−1)
Intra-B	(1 + SR_V_/N) (1 + SR_H_/N) + 2	(N + SR_H_) × (N − 1)
Intra-C	(1 + SR_V_/N) + 2	(SR_H_ + N − 1) × (SR_V_ + N − 1)
Inter-C	2 + SR_V_/N + 1/m	(SR_H_ + N − 1) × (SR_V_ + N − 1) × m
Intra-C+	3 + SR_V_/nN	(SR_H_ + N − 1) × (SR_V_ + nN − 1)
Inter-C+	2 + SR_V_/nN + 1/m	(SR_H_ + N − 1) × (SR_V_ + nN − 1) × m
Intra-D	3	(SR_H_ + W − 1) × (SR_V_ − 1)
Inter-D	1 + 2/m	(SR_H_ + W − 1) × (SR_V_ − 1) × m
Inter-E	1	2 × W × H
New Inter-E	1	W × H + 2 × N × W

Ra of different intra-frame data reuse levels for VC-MRME can also be calculated according to Equations (2)–(4). For example, Ra of Intra-C is calculated as follows, where r is the number of reference frames for one current frame (Table 5):

$$(1+SR_{V}/N)\times r+\frac{W\times H+W\times H}{W\times H}\;=SR_{V}/N\times r+r+2$$

**Table 5:** Ra and On-chip Memory Size for VC-MRME.

Level	Ra	On-Chip Memory Size
No reuse	SR_V_ × SR_H_ × r + 2	0
Intra-A	SR_V_(1 + SR_H_ /N) × r + 2	N × (N − 1) × r
Intra-B	(1 + SR_V_/N) (1 + SR_H_/N) × r + 2	(N + SR_H_) × (N − 1) × r
Intra-C	SR_V_/N × r + r + 2	(SR_H_ + N − 1) × (SR_V_ + N − 1) × r
Inter-C	SR_V_/N × (r/m + 1 − 1/m) + r + 1 + 1/m	Refer to Equation (6)
Intra-C+	SR_V_/nN × r + r + 2	(SR_H_ + N − 1) × (SR_V_ + nN − 1) × r
Inter-C+	SR_V_/nN × (r/m + 1 − 1/m) + r + 1 + 1/m	Refer to Equation (7)
Intra-D	r + 2	(SR_H_ + W − 1) × (SR_V_ − 1) × r
Inter-D	2r/m + 1	Refer to Equation (5)
Inter-E	1	W × H × (r + 1)
New Inter-E	1	W × H × r + 2 × N × W

### 3.2. Level E Inter-Frame Data Reuse (Inter-E)

**Figure 4:**
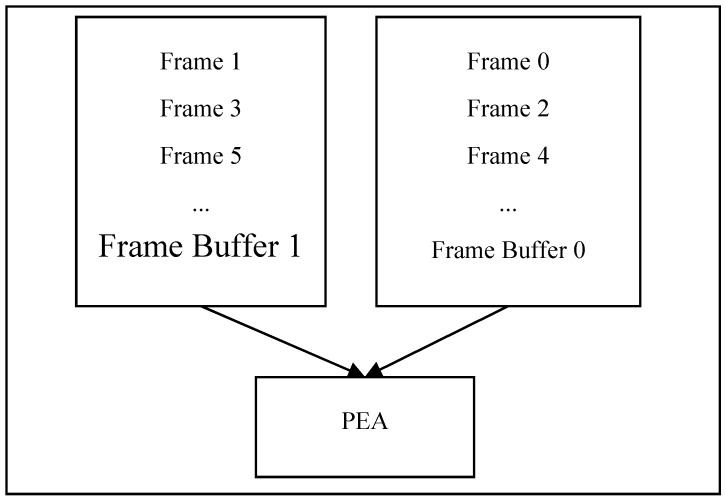
An implementation of Inter-E for FRUC-ME.

**Figure 5:**
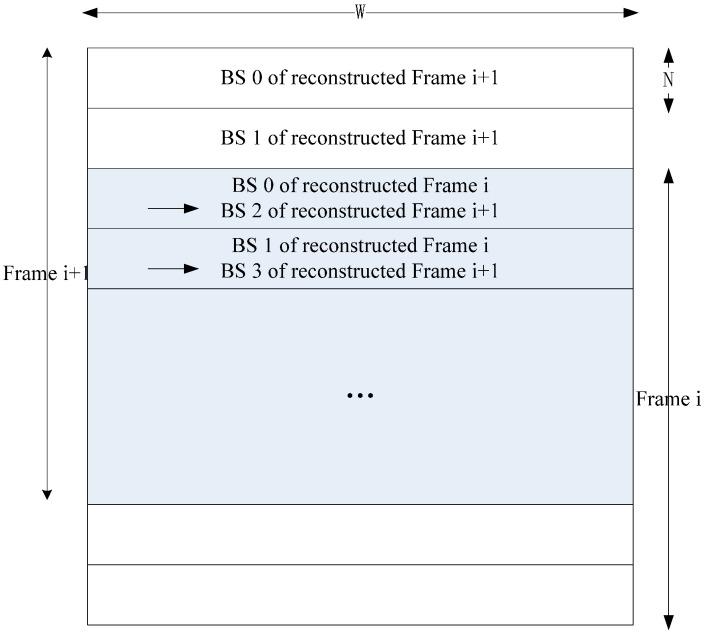
Architecture of Inter-E for VC-SRME. Circular frame buffer is used. The grey part is the shared buffer between Frame i and Frame i + 1.

To make the reconstructed frame buffered on chip and used by the next current frame, we can use two frame buffers and one PEA to implement Inter-E for VC-SRME as in Figure 4 but two frame buffers are used to store two reconstructed frames. We can reduce the size of the frame buffer by reusing the buffer between two adjacent reconstructed frames. In Figure 5, the reconstructed frame buffer is divided into H/N + 2 BS buffers and designed as a circular buffer. We assume that SRv equals 2N for convenience, which means that one SRS includes three BSs. Frame i is the reconstructed previous frame and Frame i + 1 is the reconstructed current frame. When processing BS2 of current Frame i + 1, BS1, BS2, and BS3 of reconstructed Frame i is considered as the corresponding SRS. Thus, BS0 of reconstructed Frame i is useless and can be replaced by BS2 of reconstructed Frame i + 1 (Figure 6). In the same way, BS1 of reconstructed Frame i and BS3 of reconstructed Frame i + 1 share the same BS buffer. Thus, a BS buffer is shared by two adjacent reconstructed frames. Comparing Figure 5 with Figure 4, the size of frame buffer is reduced from 2 × W × H to W × H + 2 × N × W. As only current frames are loaded from off-chip memory and reference frames are cached on chip, Ra equals 1 for Inter-E of VC-SRME.

**Figure 6:** The processing order and the BS replacement strategy of the circular frame buffer for VC_SRME.

**Table:** 

Step	Current BS in Process	Reference BS	Replaced BS in BS Buffer
0	BS0 of Frame i + 1	BS0,1 of Frame i	
1	BS1 of Frame i + 1	BS0,1,2 of Frame i	
2	BS2 of Frame i + 1	BS1,2,3 of Frame i	BS0 of Frame i
3	BS3 of Frame i + 1	BS2,3,4 of Frame i	BS1 of Frame i
4	BS4 of Frame i + 1	BS3,4,5 of Frame i	BS2 of Frame i
…	…	…	…

**Figure 7:**
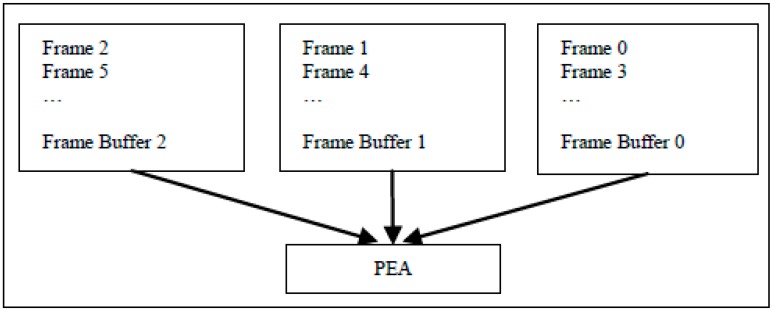
Architecture of Inter-E for VC-MRME. Three frame buffers are on chip to hold three reference frames.

**Figure 9:**
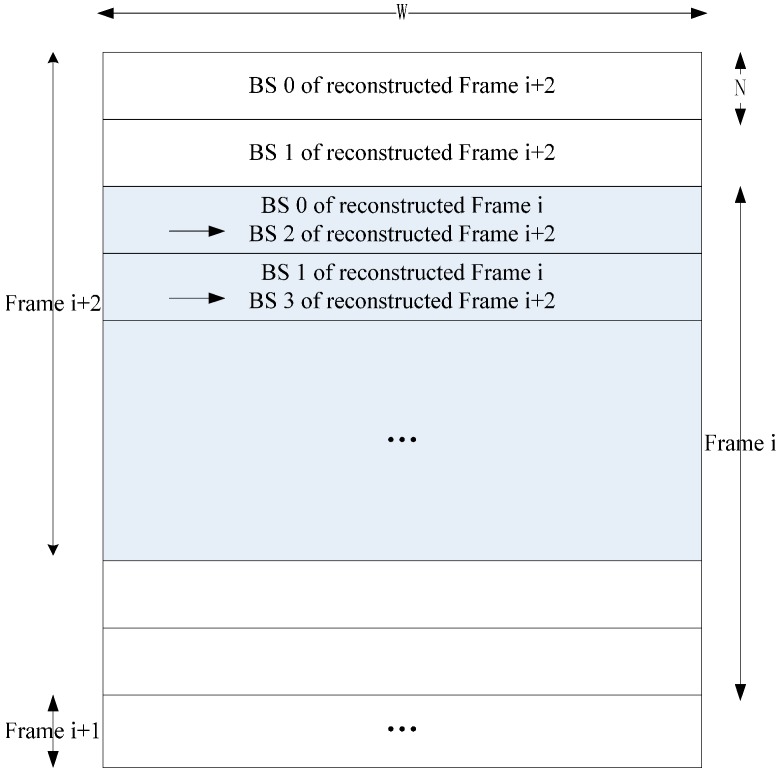
Circular frame buffer design for VC-MRME with Inter-E data reuse. The shaded part is the shared buffer between Frame i and Frame i + 2.

For VC-MRME, two reference frames are used for a current frame to explain how to implement inter-frame data reuse scheme but more reference frames are also supported. Figure 7 gives an implementation with three reference frame buffers on chip. The processing steps are shown in Figure 8. Circular buffer is used to reduce the frame buffer size (Figure 9). Frame i and Frame i + 2 share H/N − 2 BS buffers, and the frame buffer size is reduced from 3 × W × H to 2 × W × H + 2 × N × W. Ra is 1 for Inter-E of VC-MRME because only the current frame is loaded from off-chip memory and no reconstructed frame is stored to or loaded from off-chip memory.

**Figure 8:** Processing steps of the PEA for Inter-E of VC-MRME.

**Table:** 

Step	Current Frame	Reference Frame(s) in Frame Buffer for Each Step
0	Frame 1	Frame 0
1	Frame 2	Frame 0,1
2	Frame 3	Frame 1,2
3	Frame 4	Frame 2,3
…	…	…

### 3.3. Level D Inter-Frame Data Reuse (Inter-D)

**Figure 10:**
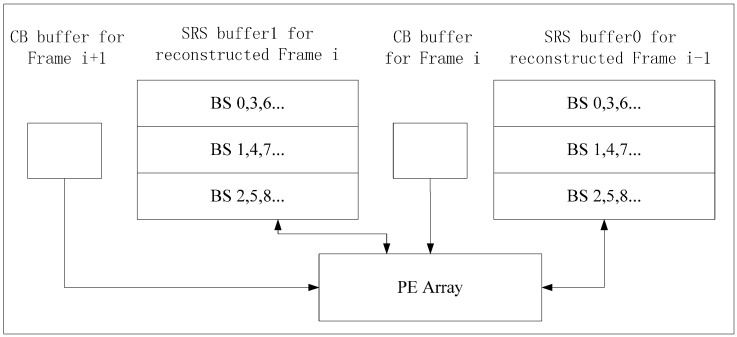
Inter-D Architecture for VC-SRME. Two SRS buffers are on-chip.

The on-chip memory size of Inter-E is at least W × H + 2 × N × W pixels, so we propose Inter-D to reduce on-chip memory size. For Inter-D, multiple SRSs of the reconstructed frame are kept on chip at the same time. An SRS buffer is used to store SRS of a reconstructed frame. In Figure 10, one PEA processes CBs of two current frames alternately in one time period, with two SRS buffers and two CB buffers (or one shared CB buffer) integrated on chip for VC-SRME. Each SRS buffer contains three BS buffers and is designed as a circular buffer. Frame i is the current frame for Frame i − 1 and the reference frame for Frame i + 1. We assume that SRV equals 2N. We try to find a way that minimum number of pixels in reconstructed Frame i are stored to or loaded from off-chip memory. In Figure 11, current BSs of Frame i and i + 1 are processed alternately with the same scan order. After processing BS2 of Frame i in Step3, reconstructed BS2 of Frame i is stored to SRS buffer1 instead of off-chip memory, and then the reconstructed BS0, BS1, and BS2 of Frame i are all in SRS buffer1 which are used as SRS for BS1 of Frame i + 1 in Step 4. In this way, reconstructed BSs of Frame i are always on chip in time for Frame i + 1 and do not need to be stored to or loaded from off-chip memory so reconstructed Frame i are inter-frame reused. However, Frame i − 1 and i + 1 cannot be inter-frame reused. There is one frame which needs to be stored to and loaded from the off-chip memory every m frames. Therefore, Ra of Inter-D for VC-SRME is calculated according to Equations (2)–(4) as follows, where m is the number of the current frames processed in one time period. When m always equals 1, it becomes Ra of Intra-D.

$$\frac{1}{m}\times \frac{W\times H}{W\times H}+\frac{1/m\times W\times H+W\times H}{W\times H}=2/m+1$$

**Figure 11:** The order of the BS in process for Inter-D of VC-SRME.

**Table:** 

Step	BS in Process	Reconstructed BS in SRS Buffer1
0	BS0 of Frame i	
1	BS1 of Frame i	BS0 of Frame i
2	BS0 of Frame i + 1	BS0,1 of Frame i
3	BS2 of Frame i	BS0,1 of Frame i
4	BS1 of Frame i + 1	BS0,1,2 of Frame i
5	BS3 of Frame i	BS0,1,2 of Frame i
6	BS2 of Frame i + 1	BS1,2,3 of Frame i
...	...	...

**Figure 12:**
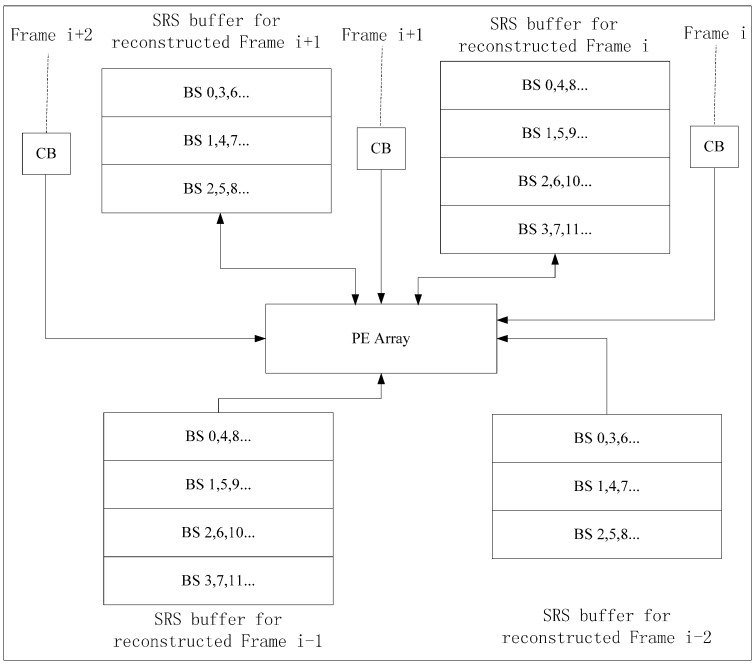
Inter-D architecture for VC-MRME. Three current frames (m = 3) are processed in the same time period, each with two reference frames (r = 2).

For VC-MRME, we also use two reference frames for one current frame to explain how to implement the inter-frame data reuse. Figure 12 gives an implementation with three current frames (Frame i, i + 1 and i + 2) processed in one time period. Three BS buffers are combined for Frame i − 2 and i + 1 to store the reconstructed SRS, and one more BS buffer is needed for reconstructed Frame i and i − 1 because these two frames are the reference frames of two current frames. The processing order of BS in the three current frames is shown in Figure 13. After one current BS of Frame i or i + 1 is processed, it is reconstructed and stored in the according BS buffer. Ra of Inter-D for VC-MRME is also calculated according to Equations (2)–(4). r is the number of reference frames for one current frame and m is the number of the current frames processed in one time period. It is assumed that m ≥ r. There are r reconstructed frames which need to be stored to and loaded from the off-chip memory every m frames, so Ra is calculated as follows.

$$\frac{r}{m}\times \frac{W\times H}{W\times H}+\frac{r/m\times W\times H+W\times H}{W\times H}=2r/m+1$$

Buffer size of Inter-D for VC-MRME is as follows (Figure 12).

(5) $$\begin{array}{l} \;\;\;(SR_{H}+W-1)(SR_{V}+N-1)\times 2\\ +(SR_{H}+W-1)(SR_{V}+2N-1)\times 2\\ +......\\ +(SR_{H}+W-1)(SR_{V}+(r-1)N-1)\times 2\\ +(SR_{H}+W-1)(SR_{V}+rN-1)\times (m-r+1) \end{array}$$

We can improve data reuse efficiency by processing more current frames in one time period and adding more SRS buffers for Inter-D of VC-ME. If there is only one SRS buffer, no reconstructed pixels can be buffered on chip and inter-frame data reuse cannot be implemented. The data reuse degree grows as the number of SRS buffers increases due to Ra reduction. For example, one *m*th of reconstructed frames are stored to off-chip memory when m SRS buffers are used for VC-SRME. In addition, Inter-D is also compatible with Intra-D for VC-ME and we can also increase parallelism by adding more PEAs.

**Figure 13:** The order of the BSs in process for Inter-D of VC-MRME.

**Table:** 

Step	Current BS in Process	Reference Pixels for the Step
0	BS0 of Frame i	BS0,1 of Frame i − 1; BS0,1 of Frame i − 2
1	BS1 of Frame i	BS0,1,2 of Frame i − 1; BS0,1,2 of Frame i − 2
2	BS0 of Frame i + 1	BS0,1 of Frame i; BS0,1 of Frame i − 1
3	BS2 of Frame i	BS1,2,3 of Frame i − 1; BS1,2,3 of Frame i − 2
4	BS1 of Frame i + 1	BS0,1,2 of Frame i; BS0,1,2 of Frame i − 1
5	BS0 of Frame i + 2	BS0,1 of Frame i + 1; BS0,1 of Frame i
6	BS3 of Frame i	BS2,3,4 of Frame i − 1; BS2,3,4 of Frame i − 2
7	BS2 of Frame i + 1	BS1,2,3 of Frame i; BS1,2,3 of Frame i − 1
8	BS1 of Frame i + 2	BS0,1,2 of Frame i + 1; BS0,1,2 of Frame i
...	...	...

### 3.4. Level C Inter-Frame Data Reuse (Inter-C)

**Figure 14:**
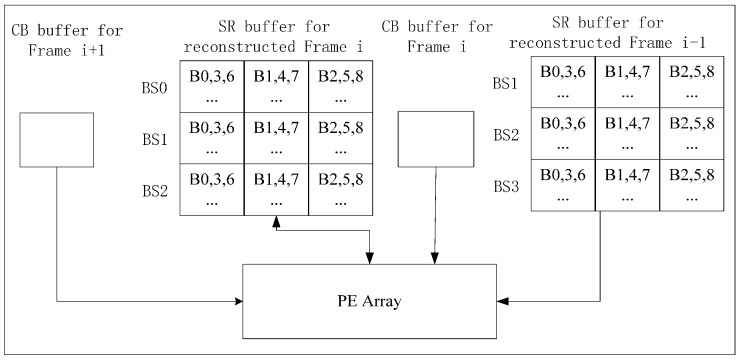
Architecture of Inter-C for VC-SRME. Two SR buffers are on chip.

We propose Inter-C to further reduce on-chip memory size. Multiple SR buffers are integrated on chip, and an SR buffer is used to store SR of a reference frame. In Figure 14, PEA processes CBs of two current frames (Frame i and i + 1) alternately for VC-SRME. Frame i is the current frame for Frame i − 1 and the reference frame for Frame i+1. Two SR buffers and two CB buffers are on chip. Both SRH and SRV equal 2N. We arrange the processing order of CBs as in Figure 15 so that part of reconstructed Frame i is kept on chip and used as SR for the CBs of Frame i + 1. Bk stands for the kth block in a BS. Bk of BSj in Frame i + 1 is processed just after Bk + 1 of BSj + 1 in Frame i. Before Step 0, B0-BS0, B1-BS0, B0-BS1 and B1-BS1 of reconstructed Frame i are already in SR buffer, and BS0, BS1 of Frame i and BS0 of Frame i + 1 have been processed. After B0, B1, and B2 of BS2-Frame i are reconstructed, the SR for B1-BS1-Frame i + 1 is produced. When processing B1-BS1-Frame i + 1 in Step 3, only B2-BS0-Frame i and B2-BS1-Frame i are loaded from off-chip memory and B2-BS2-Frame i is already in the SR buffer. In this way, the reconstructed data of Frame i are partly inter-frame reused in the SR buffer. However, Frame i − 1 and i + 1 are only intra-frame reused. A part of reference frame is kept on chip for reuse, so Ra_intra of Inter-C is calculated as follows.

$$\frac{H/N\times (W+SR_{H})\times N\times 1/m+H/N\times (W+SR_{H})\times SR_{V}}{W\times H}\approx 1/m+SR_{V}/N$$

Every reconstructed frame should be stored to off-chip memory so Ra_inter of Inter-C equals 2. Then Ra of Inter-C is 2 + SR_V_/N + 1/m. When m equals 1, it becomes Ra of Intra-C.

**Figure 15:** The order of the CBs in process for Inter-C of VC-SRME.

**Table:** 

Step	CB in Process	Data in SR Buffer for Reconstructed Frame i	Data from Off-Chip Memory
0	B0-BS2-Frame i	B0-BS0,B1-BS0 B0-BS1,B1-BS1	B1-BS1-Frame i − 1
			B1-BS2-Frame i − 1
			B1-BS3-Frame i − 1
1	B1-BS2-Frame i	B0-BS0,B1-BS0	B2-BS1-Frame i − 1
		B0-BS1,B1-BS1	B2-BS2-Frame i − 1
		B0-BS2	B2-BS3-Frame i − 1
2	B2-BS2-Frame i	B0-BS0,B1-BS0	B3-BS1-Frame i − 1
		B0-BS1,B1-BS1	B3-BS2-Frame i − 1
		B0-BS2,B1-BS2	B3-BS3-Frame i − 1
3	B1-BS1-Frame i + 1	B0-BS0,B1-BS0	B2-BS0-Frame i B2-BS1-Frame i
		B0-BS1,B1-BS1	
		B0-BS2,B1-BS2,B2-BS2	
4	B3-BS2-Frame i	B1-BS0,B2-BS0	B4-BS1-Frame i − 1
		B1-BS1,B2-BS1	B4-BS2-Frame i − 1
		B1-BS2,B2-BS2	B4-BS3-Frame i − 1
5	B2-BS1-Frame i + 1	B1-BS0,B2-BS0	B3-BS0-Frame i B3-BS1-Frame i
		B1-BS1,B2-BS1	
		B1-BS2,B2-BS2,B3-BS2	
6	B4-BS2-Frame i	B2-BS0,B3-BS0	B5-BS1-Frame i − 1
		B2-BS1,B3-BS1	B5-BS2-Frame i − 1
		B2-BS2,B3-BS2	B5-BS3-Frame i − 1
7	B3-BS1-Frame i + 1	B2-BS0,B3-BS0	B4-BS0-Frame i B4-BS1-Frame i
		B2-BS1,B3-BS1	
		B2-BS2,B3-BS2,B4-BS2	
...	...	…	...

**Figure 16:**
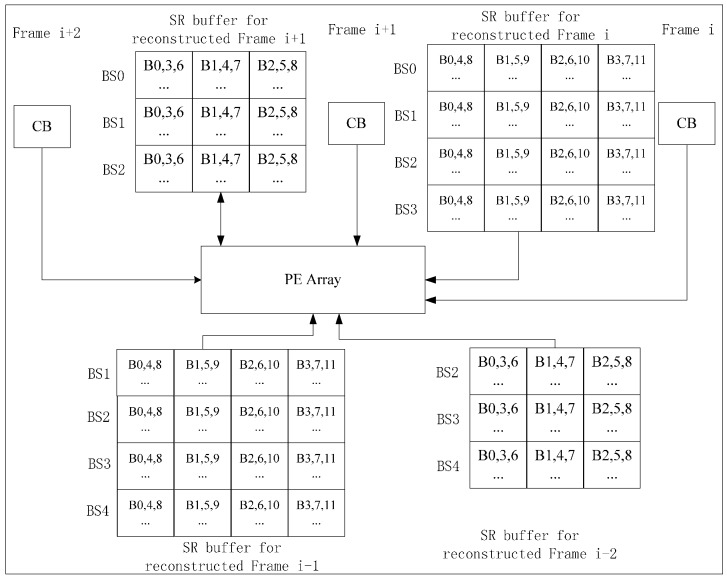
Architecture of Inter-C for VC-MRME.

For VC-MRME, we use two reference frames for one current frame to explain how to implement Inter-C. Figure 16 gives an implementation with three current frames processed in one time period, Frame i, i + 1 and i + 2. Part of reconstructed Frame i − 2, i − 1, i, and i + 1 are buffered on chip as SR for current frames. Bk in BSj of Frame i + 1 is processed just after Bk + 1 in BSj + 1 of Frame i, and Bk − 1 in BSj − 1 of Frame i + 2 is processed just after Bk in BSj of Frame i + 1 (Figure 17). Step 0 is an initial step to load twelve N × N blocks of two reference frames for B0-BS3-Frame i. Step 2 and 5 are initial steps to load six N × N blocks of two reference frames for B0-BS2-Frame i + 1 and B0-BS1-Frame i + 2 respectively. After the initial steps, only six N × N blocks of two reference frames are loaded for each CB of Frame i and only three N × N blocks of two reference frames are loaded for each CB of Frame i + 1 and i + 2 . r is the number of reference frames for one current frame and m is the number of the current frames processed in one time period. It is assumed that m ≥ r. A part of the reference frames is kept on chip for reuse. Every m frames, there is one frame(Frame i in Figure 16) which needs to load H/N × (W + SR_H_) × (N + SR_V_) × r pixels, and the other m-1 frames only needs to load H/N × (W + SR_H_) × ((r−1)N + SR_V_) pixels. So Ra_intra of Inter-C is calculated as follows. When r equals 1, the Ra_intra is the same as that of VC-SRME.

$$\begin{array}{l} \frac{1}{m}\times \frac{H/N\times (W+SR_{H})\times (N+SR_{V})\times r+H/N\times (W+SR_{H})\times (SR_{V}+(r-1)N)\times (m-1)}{W\times H}\\ =SR_{V}/N\times (r/m+1-1/m)+r-1+1/m \end{array}$$

All the data of the reconstructed frame should be stored to off-chip memory and Ra_inter of Inter-C equals 2. Then Ra of Inter-C is as follows:

$$SR_{V}/N\times (r/m+1-1/m)+r+1+1/m$$

The buffer size of Inter-C for VC-MRME is as follows:

(6) $$\begin{array}{l} \;\;\;(SR_{H}+N-1)(SR_{V}+N-1)\times 2\\ +(SR_{H}+2N-1)(SR_{V}+2N-1)\times 2\\ +......\\ +(SR_{H}+rN-1)(SR_{V}+rN-1)\times (m-r+1) \end{array}$$

Calculated in the same way as Inter-C, the buffer size of Inter-C+ for VC-MRME is as follows:

(7) $$\begin{array}{l} \;\;\;(SR_{H}+N-1)(SR_{V}+nN-1)\times 2\\ +(SR_{H}+2N-1)(SR_{V}+(n+1)N-1)\times 2\\ +......\\ +(SR_{H}+rN-1)(SR_{V}+(n+r-1)N-1)\times (m-r+1) \end{array}$$

**Figure 17:** The order of the CBs in process for Inter-C of VC-MRME. The shaded blocks with block number are the blocks which need to be loaded in the current step, the blank blocks are the empty buffers, and the white blocks with block number are the blocks already in the buffer.

**Table:** 

Step	CB in Process	SR buffer i+1	SR Buffer i	SR Buffer i−1	SR Buffer i−2
0	B0-BS3-Frame i	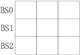	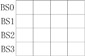	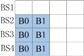	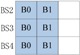
1	B1-BS3-Frame i	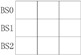	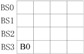	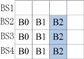	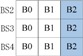
2	B0-BS2-Frame i + 1	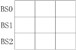	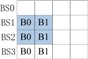	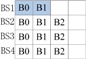	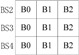
3	B2-BS3-Frame i	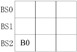	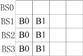	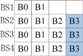	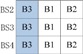
4	B1-BS2-Frame i + 1	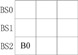	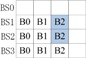	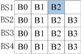	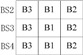
5	B0-BS1-Frame i + 2	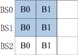	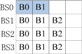	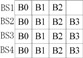	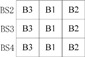
6	B3-BS3-Frame i	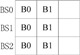	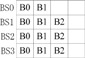	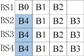	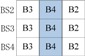
7	B2-BS2-Frame i + 1	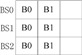	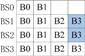	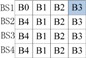	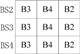
8	B1-BS1-Frame i + 2	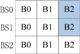	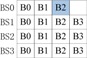	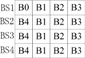	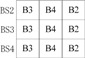
...	...	…	…	…	…

We can also improve data reuse efficiency by adding more SR buffers and increase parallelism by adding more PEAs for VC-ME. The data reuse degree grows as the number of current frames processed in one time period increases. In addition, Inter-C (Inter-C+) is compatible with Intra-C (Intra-C+).

### 3.5. Analysis and Comparison of Different Data Reuse Levels

We list *Ra* and on-chip memory requirement for different reuse levels of VC-SRME in Table 4. *m* is the number of the SR or SRS buffers on chip. n is the parameter for C+ scheme. The proposed inter-frame data reuse schemes always have better reuse efficiency than their intra-frame counterparts. A larger *m* leads to a smaller *Ra* but a larger on-chip memory size. *Ra* of Inter-D is less than half of *Ra* of Intra-D when *m* is greater than 2 but the on-chip memory size of Inter-D is *m* times of Intra-D. Comparing with Inter-E, the on-chip memory size of Inter-D and Inter-C are reduced to *m* SRS buffers and *m* SR buffers respectively. We give *Ra* and on-chip memory size for different reuse levels of VC-MRME in Table 5. m is the number of the SR or SRS buffers on chip. n is the parameter for C+ scheme. r is the number of reference frames for one current frame. The proposed inter-frame data reuse schemes also show better reuse efficiency than their intra-frame counterparts when *m* ≥ *r* ≥ *2*.

## 4. Implementation of Inter-D for VC-SRME

**Figure 18:**
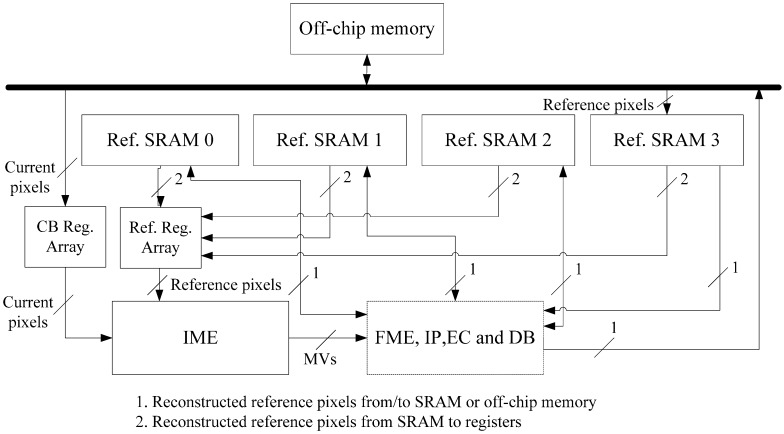
The implemented architecture for Inter-D VC-SRME.

**Figure 19:**
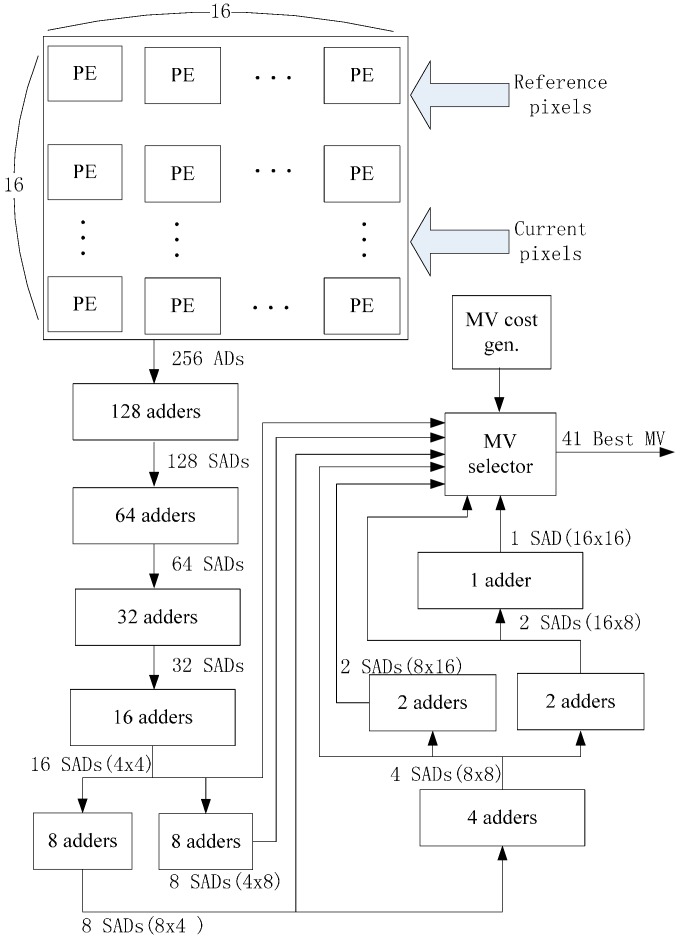
The implemented architecture for IME.

Inter-D gives a useful tradeoff between off-chip memory traffic and on-chip memory size so we implement an Inter-D architecture for VC-SRME (Figure 18) with *m* = 4. The IME module in this implementation (Figure 19) mainly comprises of one SAD Tree [15] with a MV selector (41 SAD comparators), which can support variable block size ME. FME, IP, EC and DB are the other modules for a complete encoder architecture but these modules are not included in our implementation. Fractional ME (FME) is usually the module after IME in an encoding pipeline. Instead of having its own on-chip buffer, FME can load data directly from IME buffer [31], so the proposed data reuse scheme is compatible with FME. CB Reg. Array is a 16 × 16 register array which is used to store the current block. A two-level memory hierarchy is implemented between IME and off-chip memory for loading reference pixels. The first level is a 16 × 16 Ref. Reg. Array which employs Intra-A. The Ref. Reg. Array receives reference pixels from four Ref. SRAMs alternately as described in Figure 11. The second level is four Ref. SRAMs which are used to employ Inter-D. Only Ref. SRAM 3 receives reference pixels from off-chip memory and other Ref. SRAMs all receive reference pixels which are produced on chip to reduce the off-chip memory traffic.

**Figure 20:**
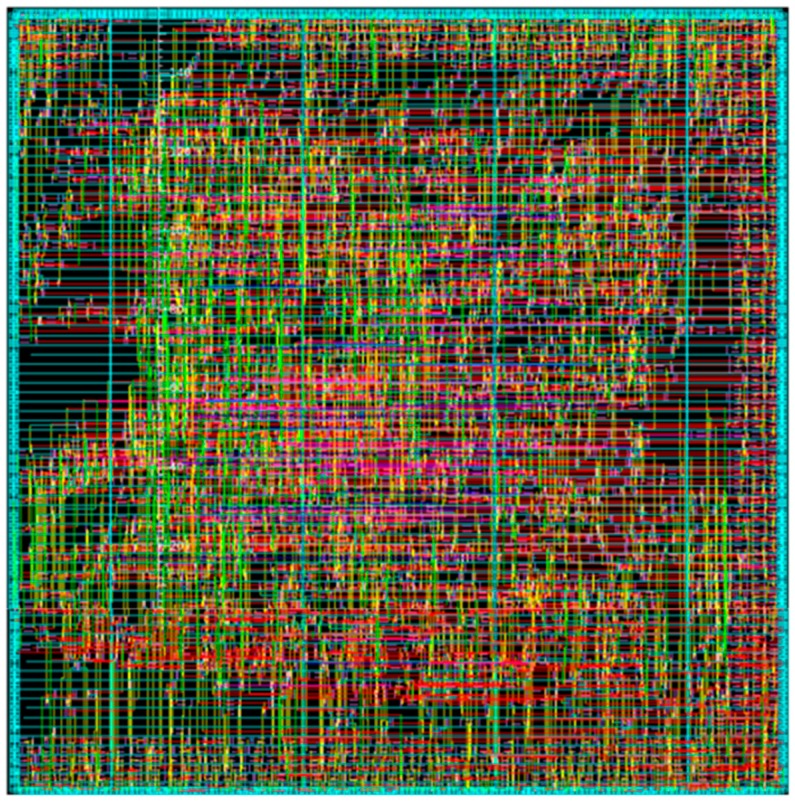
Picture of the implemented ME module in 65 nm.

We implement the Inter-D VC-SRME architecture with synthesizable Verilog HDL and list synthesis results using a TSMC 65 GP technology with 360 MHz (Table 6). Figure 20 gives the picture of the implemented ME module in 65 nm. The proposed architecture is compared with other FSIME architectures using intra-frame data reuse. All the works in Table 6 use full search (FS) with the frame rate of 30 f/s and the block size of 16 × 16 but they support different resolutions, SR or numbers of reference frames. The reuse level (off-chip to on-chip) affects the off-chip memory bandwidth requirement. Our implementation adopts Inter-D data reuse so it achieves the best off-chip memory access efficiency. However, the off-chip bandwidth is not only related with the data reuse level (Inter-D in the proposed architecture or Intra-D in [29]) but also related with other parameters such as resolution, frame rate, SR, N and so on. Some of these parameters are different between [29] and our work so the off-chip bandwidth of our work (Inter-D) is greater than that of [29] (Intra-D). If the parameters are all the same for the two works, Inter-D will only need half the off-chip memory traffic of Intra-D. In Section 5, we will give the comparison between the proposed Inter-D and Intra-D [29] under the same parameters. The reuse level (on-chip) represents the reuse level from SRAM to registers on chip and affects on-chip memory bandwidth requirement. The reference [32] proposed a novel data reuse method “Inter-candidate + Inter-macroblock” to reduce the on-chip memory bandwidth to 5 GByte/s. This method can be regarded as improved Intra-A or Inter-A. However, the goal of our proposed method is to reduce the off-chip memory traffic which is usually the performance bottleneck. The proposed inter-frame data reuse methods in this paper are compatible with “Inter-candidate + Inter-macroblock”. So we simply use a Level A data reuse with “Inter-candidate + Inter-macroblock” for reducing on-chip memory bandwidth. Note that some implementations [25,29] in Table 6 only consider the memory traffic of loading the reference frame when computing off-chip or on-chip bandwidth requirement and neglect the memory traffic of loading current frames or storing reference frames. Both Level A parallelism and Level B parallelism (eight SAD Tree) are implemented in [25] while only Level A parallelism (one SAD Tree) is implemented in our design. The gate count increases as the parallelism level increases because more PEAs are used. Due to the fact we use an Inter-D data reuse, the implementation demands a larger on-chip SRAM size (241.92 KB) than the other three works.

**Table 6:** Comparison between Different FSIME Implementations.

	This Work	[29]	[25]	[32]
Search method	FS	FS	FS	FS
Resolution	1920 × 1080	720 × 576	720 × 480	1920 × 1088
Frame rate	30 fps	30 fps	30 fps	30 fps
Reference frames	1	2	4	1
SR	32 × 32	65 × 33	128 × 64, 64 × 32	32 × 32
N	16	16	16	16
Reuse level(off-chip to on-chip)	Inter-D	Intra-D	Intra-C	Intra-C
Reuse level (on-chip)	Intra-A	Intra-A	Intra-A	Intra-A
Parallelism level	A	A	A,B	A,B,C
Off-chip bandwidth (MByte/s)	93.31	24.9	135.9	319
On-chip bandwidth (MByte/s)	5120	-	-	5120
Technology	65 nm	0.18 um	0.18 um	0.18 um
Frequency (MHz)	360	216	81	130
Gate count (K)	166	168	330	1449
SRAM (KB)	241.92	23.75	26	1.26

## 5. Experiment Results

We give three case studies (1080 p, 720 p and 4 K) to analyze and compare different data reuse levels for VC-ME. Ra and on-chip memory size of two different application scenarios are computed according to Table 4 and Table 5 respectively, where m and n both equals 4. Intra-A, Intra-B, Intra-C, Intra-D, and Inter-E are traditional data reuse methods. Inter-A, Inter-B, Inter-C, Inter-D and New Inter-E are proposed inter-frame data reuse methods. Bandwidth in Equation (8) is the off-chip memory bandwidth requirement. f is frame rate.

(8) Bandwidth=f×W×H×Ra

For VC-SRME (Table 7), the bandwidth requirement reductions of Inter-D are 50% compared with Intra-D [29], which is the largest memory traffic reduction ratio for VC-SRME. Inter-E needs 4.1 MB on-chip memory for 1080 p, 1.8 MB for 720 p and 16.6 MB for 4 K, and the on-chip memory size is reduced nearly by half after using the proposed circular frame buffer (New Inter-E) while the bandwidth requirement is the same. Compared with no data reuse, Intra-A [20] and Intra-B [20] can reduce a large amount of memory traffic with a small on-chip memory size. However, they still demand too much memory bandwidth. The proposed three inter-frame data reuse schemes all have better data reuse efficiency than their intra-frame counterparts for all the three specifications. For 1080p and 4K, the bandwidth requirement reductions of Inter-C, Inter-C+, and Inter-D are 15%, 21.4%, and 50% respectively, compared with Intra-C [25,29], Intra-C+ [21], and Intra-D. For 720 p, the bandwidth requirement reductions of Inter-C, Inter-C+, and Inter-D are 18.8%, 23.1%, and 50% respectively, compared with Intra-C, Intra-C+, and Intra-D.

**Table 7:** Three Case Studies for VC-SRME.

Reuse Level	1080 p, 30 fps,			720 p, 30 fps,			4 K, 60 fps,		
	SR_H_ = SR_V_ = 32, *N* = 16			SR_H_ = SR_V_ = 16, *N* = 16			SR_H_ = SR_V_ = 128, *N* = 64		
	Ra	Bandwidth (MByte/s)	On-Chip Memory Size (KB)	Ra	Bandwidth (MByte/s)	On-Chip Memory Size (KB)	Ra	Bandwidth (MByte/s)	On-Chip Memory Size (KB)
No reuse	1026	63,825.4	0	258	7133.2	0	16,386	8,154,722.3	0
Intra-A	98	6096.4	0.24	34	940.0	0.24	386	192,098.3	4.03
Intra-B	11	684.3	0.72	6	165.9	0.48	11	5474.3	12.10
Intra-C	5	311.0	2.21	4	110.6	0.96	5	2488.3	36.48
Inter-C	4.25	264.4	8.84	3.25	89.9	3.84	4.25	2115.1	145.92
Intra-C+	3.5	217.7	4.47	3.25	89.9	2.45	3.5	1741.8	73.15
Inter-C+	2.75	171.1	17.86	2.5	69.1	9.80	2.75	1368.6	292.61
Intra-D	3	186.6	60.48	3	82.9	19.43	3	1493.0	503.81
Inter-D	1.5	93.3	241.92	1.5	41.5	77.72	1.5	746.5	2015.24
Inter-E	1	62.2	4147.20	1	27.7	1843.20	1	497.7	16,588.80
New Inter-E	1	62.2	2135.04	1	27.7	962.56	1	497.7	8785.92

For VC-MRME (Table 8), the video sequence is encoded with one I frame initially and the following frames are all encoded using the previously encoded and reconstructed r frames. r equals 4 in this case study. The on-chip memory size is r times of VC-SRME for the corresponding intra-frame data reuse level because it has to keep r buffers for r reference frames. Compared with no data reuse, Intra-A and Intra-B can reduce a large amount of memory traffic with a small on-chip memory size. However, they still demand too much memory bandwidth, 24.0 GByte/s and 2.4 GByte/sec for Intra-A and Intra-B, respectively. For 1080 p and 4 K, the bandwidth requirement reductions of Inter-C, Inter-C+, and Inter-D are 37.5%, 30.6%, and 50% respectively, compared with their intra-frame counterparts. For 720 p, the bandwidth requirement reductions of Inter-C, Inter-C+, and Inter-D are 30%, 18.8%, and 50% respectively, compared with their intra-frame counterparts.

**Figure 21:**
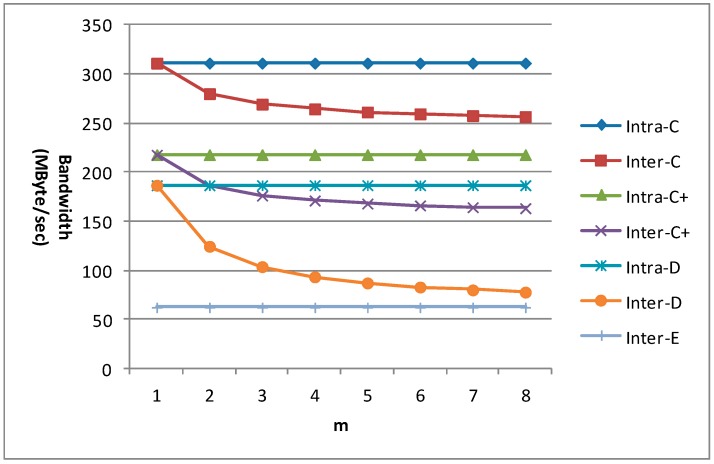
The bandwidth requirement of VC-SRME for 1080 p.

*m* is an important factor which affects the data reuse efficiency for the new inter-frame data reuse scheme. So the bandwidth requirement for 1080 p with different m is given in Figure 21 for different data reuse levels of VC-SRME. n equals 4 in the C+ scheme. We can find that the bandwidth requirements of all the intra-frame data reuse levels (including Intra-A and Intra-B which are not shown in the figure) and Inter-E are unchanged with the variation of m. When m equals 1, the bandwidth requirement of the inter-frame data reuse level is the same as that of its corresponding intra-frame data reuse level. The bandwidth requirements of Inter-C, Inter-C+, and Inter-D become lower with the increase of m, but the bandwidth reduction magnitude becomes smaller because there is always a constant which will not change with m in the formula of Ra. We find that the bandwidth requirement reduction for Inter-D is more effective than that for Inter-C or Inter-C+ because Inter-D can reuse the reconstructed frame more efficiently.

**Table 8:** Three Case Studies for VC-MRME.

Reuse Level	1080 p, 30 fps, SR_H_ = SR_V_ = 32, *N* = 16			720 p, 30 fps, SR_H_ = SR_V_ = 16, *N* = 16			4 K, 60 fps, SR_H_ = SR_V_ = 128, *N* = 64		
	Ra	Bandwidth (MByte/s)	On-Chip Memory Size(KB)	Ra	Bandwidth (MByte/s)	On-Chip Memory Size(KB)	Ra	Bandwidth (MByte/s)	On-Chip Memory Size(KB)
No reuse	4098	254,928.4	0	1026	28,366.9	0	65538	32,615,903.2	0
Intra-A	386	24,012.3	0.96	130	3594.2	0.96	1538	765,407.2	16.13
Intra-B	38	2363.9	2.88	18	497.7	1.92	38	18,911.2	48.38
Intra-C	14	870.9	8.84	10	276.5	3.84	14	6967.3	145.92
Inter-C	8.75	544.3	33.86	7	193.5	20.52	8.75	4354. 6	553.22
Intra-C+	8	497.7	17.86	7	193.5	9.80	8	3981.3	292.61
Inter-C+	6.125	381.0	56.57	5.6875	157.3	37.85	6.125	3048.2	920.52
Intra-D	6	373.3	241.92	6	165.9	77.70	6	2986.0	2015.24
Inter-D	3	186.6	922.82	3	82.9	467.50	3	1493.0	7588.87
Inter-E	1	62.2	10,368.00	1	27.7	4608.00	1	497.7	41,472.00
New Inter-E	1	62.2	8355.84	1	27.7	3727.36	1	497.7	33,669.12

**Figure 22:**
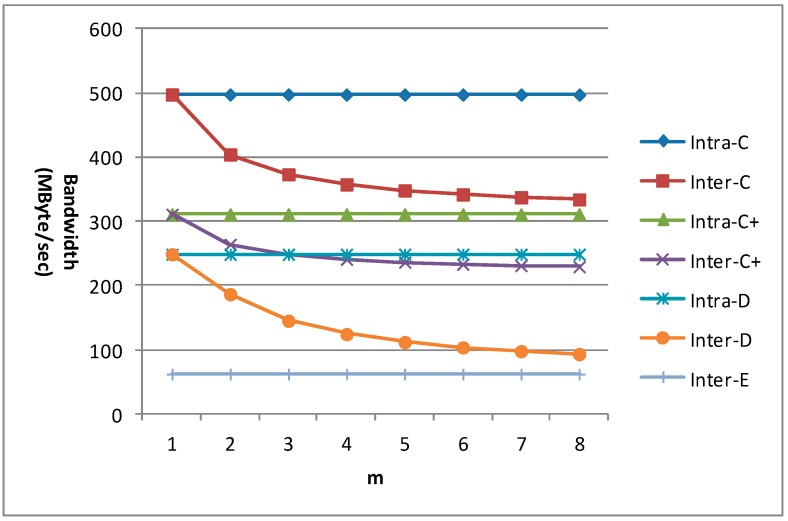
The bandwidth requirement of VC-MRME for 1080 p.

The bandwidth requirement for 1080 p with different m is given in Figure 22 for different data reuse levels of VC-MRME. n equals 4 in the C+ scheme and r equals 2. We can find that the bandwidth requirements of all the intra-frame data reuse levels (including Intra-A and Intra-B which are not shown in the figure) and Inter-E are unchanged with the variation of m. When m equals 1, the bandwidth requirement of the inter-frame data reuse level is the same as that of its corresponding intra-frame data reuse level. The bandwidth requirements of Inter-C, Inter-C+, and Inter-D become lower with the increase of m, but the bandwidth reduction magnitude becomes smaller because there is always a constant which will not change with m in the formula of Ra.

**Figure 23:**
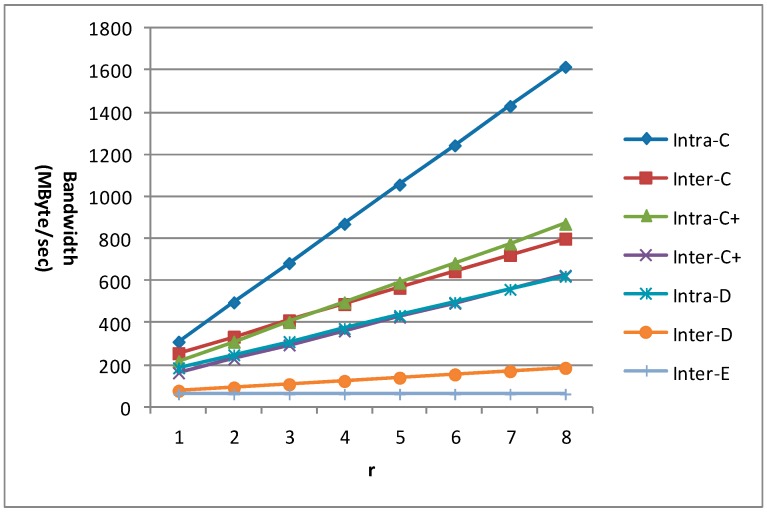
The bandwidth requirement of VC-MRME for 1080 p with different values of r. n equals 4 for C+ scheme. *m* equals 8.

The value of r is a factor which can reflect how the number of reference frames affects the memory traffic of different data reuse levels for VC-MRME, so we give the bandwidth requirements of different values of r when n equals 4 and m equals 8 in Figure 23. There is a linear relationship between the value of r and the bandwidth requirements of different data reuse levels, which means that a larger r will lead to more memory traffic because more data need to be loaded from off-chip memory. The slope of an intra-frame data reuse scheme is larger than that of its inter-frame counterpart, which means that memory traffic of the intra-frame data reuse increases faster with r than its inter-frame counterpart. Therefore, the proposed inter-frame data reuse scheme is more scalable than the traditional intra-frame data reuse scheme for multiple reference frames ME.

**Figure 24:**
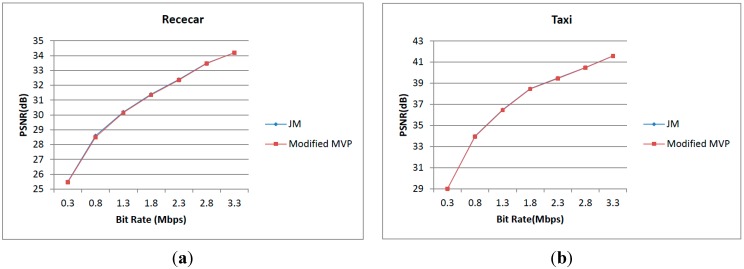
Comparison of RD curves between JM and modified motion vector prediction. (**a**) The comparison between JM and modified motion vector prediction in Racecar; (**b**) The comparison between JM and modified motion vector prediction in Taxi.

The Level C parallelism defined in Section 2 is based on modifying the calculation of the predicted motion vector (MVP) [25]. Therefore, the quality analysis is needed to be accomplished. Figure 24a,b shows the comparisons of RD curves between JM and the modified motion vector prediction. Many sequences have been tested from QCIF to HDTV, and two of them are Racecar (720 × 288, 30 fps) and Taxi (672 × 288, 30 fps). From the figure, we can find that the quality loss is near zero at high bit rates (larger than 1 Mb/s) and the quality is degraded 0.1 dB at a low bit rate [25]. We can conclude that the coding performance of the modified MVP is competitive with that of JM. For Level A and B parallelism and the proposed different data reuse levels, a standard motion vector prediction and a classical full search algorithm is used and nothing which can affect quality is modified. Therefore, the best quality can be achieved.

Our method can effectively reduce the number of off-chip memory accesses, which will affect positively the power consumption. We perform power consumption analysis for different data reuse schemes, Intra-C, Inter-C, Intra-C+, Inter-C+, Intra-D, and Inter-D. We model the DRAM access power according to [33,34]. We assume that the static power is constant with respect to accesses and evenly distributed across all banks. The dynamic power is proportional to the read bandwidth and write bandwidth. The equation for DRAM access power is as follows [34], where BWr and BWw represent the read bandwidth and write bandwidth in GB/s. Table 9 gives the power consumptions of the 1080 p case for VC-SRME according to Table 7. We find that the power consumptions of the inter-frame data reuse schemes are all lower than their intra-frame counterparts.

P (mW)=5.85+753 × BWr+671 × BWw

**Table 9:** Power analysis of 1080 p for VC-SRME.

	BWr (GB/s)	BWw (GB/s)	P (mW)
Intra-C	0.249	0.062	234.95
Inter-C	0.202	0.062	199.56
Intra-C+	0.156	0.062	164.92
Inter-C+	0.109	0.062	129.53
Intra-D	0.124	0.062	140.82
Inter-D	0.031	0.062	70.80

## 6. Conclusions

In this paper, we propose a novel inter-frame data reuse scheme for FSIME in image sensors with video compression. The new scheme improves data reuse efficiency of the reconstructed frame for VC-ME. The proposed inter-frame data reuse scheme effectively reduces the number of off-chip memory accesses and outperforms the traditional intra-frame scheme on memory bandwidth requirement.
